# Action-Effect Bindings and Ideomotor Learning in Intention- and Stimulus-Based Actions

**DOI:** 10.3389/fpsyg.2012.00444

**Published:** 2012-10-25

**Authors:** Arvid Herwig, Florian Waszak

**Affiliations:** ^1^Department of Psychology, Bielefeld UniversityBielefeld, Germany; ^2^Department of Psychology, Max Planck Institute for Human Cognitive and Brain SciencesLeipzig, Germany; ^3^Université Paris Descartes, Sorbonne Paris CitéParis, France; ^4^CNRS, Laboratoire Psychologie de la Perception, UMR 8158Paris, France

**Keywords:** feature binding, event file, action-effect, sensorimotor integration, ideomotor learning

## Abstract

According to ideomotor theory, action-effect associations are crucial for voluntary action control. Recently, a number of studies started to investigate the conditions that mediate the acquisition and application of action-effect associations by comparing actions carried out in response to exogenous stimuli (stimulus-based) with actions selected endogenously (intention-based). There is evidence that the acquisition and/or application of action-effect associations is boosted when acting in an intention-based action mode. For instance, bidirectional action-effect associations were diagnosed in a forced choice test phase if participants previously experienced action-effect couplings in an intention-based but not in a stimulus-based action mode. The present study aims at investigating effects of the action mode on action-effect associations in more detail. In a series of experiments, we compared the strength and durability of short-term action-effect associations (binding) immediately following intention- as well as stimulus-based actions. Moreover, long-term action-effect associations (learning) were assessed in a subsequent test phase. Our results show short-term action-effect associations of equal strength and durability for both action modes. However, replicating previous results, long-term associations were observed only following intention-based actions. These findings indicate that the effect of the action mode on long-term associations cannot merely be a result of accumulated short-term action-effect bindings. Instead, only those episodic bindings are selectively perpetuated and retrieved that integrate action-relevant aspects of the processing event, i.e., in case of intention-based actions, the link between action and ensuing effect.

## Introduction

Humans either carry out actions to achieve desired effects in the environment or to accommodate to environmental demands. For instance, pressing the cappuccino button on a coffee dispenser is primarily based on the agent’s intention to have a hot cup of coffee. In contrast, flooring the brake pedal at a red traffic light is chiefly performed in response to a prior stimulus event. These two types of action have been labeled voluntary, operant, or *intention-based*, on the one side, and reaction, response, or *stimulus-based*, on the other side.

Neuroscientific evidence suggests that intention- and stimulus-based actions have distinct neural bases (e.g., Goldberg, 1985; Passingham, 1993; Praamstra et al., 1995; Deiber et al., 1996; Waszak et al., 2005, 2012; Mueller et al., 2007; Haggard, 2008). This distinction is further supported by clinical observations showing a selective impairment of one type of action and thereby implying dissociation between intention- and stimulus-based action control (e.g., Lhermitte, 1983; Shallice et al., 1989).

However, the actual processes that guide these two types of actions are still not well understood. One obvious functional difference between intention- and stimulus-based actions is the role of external stimuli either preceding or following the action. According to ideomotor reasoning (e.g., Harleß, 1861; James, 1890; for recent reviews, see Nattkemper et al., 2010; Shin et al., 2010; Pfister and Janczyk, 2012) intention-based actions are primarily directed at and selected by the effects following the action whereas there is a less obvious connection to preceding stimuli. On the contrary, stimulus-based actions, by definition, crucially depend on preceding stimuli whereas stimuli following the action are often less important. Thus, it has been suggested that intention-based actions rely more strongly on action-effect associations specifying which action produces which effect, whereas stimulus-based actions rely more strongly on stimulus-response associations specifying which motor routines action-relevant stimuli habitually require (Waszak et al., 2005, 2012; Herwig et al., 2007, 2013; Pfister et al., 2010).

### Ideomotor learning

The purported functional difference is supported by a number of recent studies directly comparing the long-term consequences of actions carried out in response to exogenous stimuli (stimulus-based) with actions selected endogenously (intention-based). For instance, Herwig et al. (2007) investigated ideomotor learning, that is, the spontaneous acquisition of action-effect associations, in intention- and stimulus-based actions. Ideomotor learning can be assessed in a paradigm conceived by Elsner and Hommel (2001). These authors made participants first undergo an acquisition phase, in which a self selected key press always produced a particular tone (e.g., left key press/high-pitch tone; right key press/low-pitch tone). After having performed about 200 key presses, the same tones were presented as imperative stimuli for a speeded choice response in a subsequent test phase. Elsner and Hommel observed that the speeded choice responses were faster in response to the tone that the action had previously produced (e.g., compatible group: low-pitch tone/right key press) than to a tone that had been produced by the alternative action (e.g., incompatible group: high-pitch tone/right key press). This result demonstrates that, during the acquisition phase, participants acquire long-lasting bidirectional associations between the motor code of the action and the perceptual code of the auditory effect (i.e., action-effect associations). Presenting the effects as imperative stimuli in a later test phase leads to the retrieval of the previously acquired action-effect association which either speeds up or slows down the speeded choice task depending on whether the retrieved actions are compatible or incompatible with the instructed response.

Importantly, the effect of action-effect associations on a subsequent speeded choice task depends on the action mode during acquisition (Herwig et al., 2007; Herwig and Waszak, 2009; Herwig and Horstmann, 2011; but, see Pfister et al., 2011, for different results with a free choice test). That is, in the studies of Herwig and colleagues, a compatibility effect only occurred if, in the acquisition phase, participants freely selected between left and right key presses (intention-based acquisition), whereas there was no compatibility effect if the actions were triggered by external stimulus events (stimulus-based acquisition). This dependency on the action mode holds true for such different effect- and action-modalities like auditory effects and manual actions (Herwig et al., 2007; Herwig and Waszak, 2009) as well as visual effects and oculomotor actions (Herwig and Horstmann, 2011). Moreover, guiding participants’ attention away or toward the effect did not influence the pattern of results (Herwig and Waszak, 2009). Thus, the observed differences between intention- and stimulus-based actions are not simply due to differences in allocation of attention to the action-effect event. Instead, the results suggest that one and the same action-effect event results in different long-term consequences depending on the action mode: if actions are performed in the intention-based mode, ideomotor learning occurs, that is new action-effect associations are acquired and later on retrieved upon effect presentation. In contrast, if actions are selected in the stimulus-based mode, sensorimotor learning occurs, that is stimulus-response associations are established while action-effect associations are much harder to detect subsequently.

It has to be noted that to date it is still under debate why action-effect associations are much harder to detect following stimulus-based actions and different hypotheses have been proposed. Herwig et al. (2007) suggested that the action mode affects the acquisition of action-effect associations. Accordingly, action-effect associations are weaker following a stimulus-based acquisition compared to an intention-based acquisition which in turn hampers their later detection. However, the different-acquisition hypothesis was recently put into question by two studies showing ideomotor learning also following stimulus-based actions (Pfister et al., 2011; Wolfensteller and Ruge, 2011). In the study of Pfister et al. (2011) ideomotor learning was assessed in a free choice test phase, in which participants were presented with randomly selected action-effects, which merely served as a trigger to carry out a self-chosen response. Under these test conditions participants preferred the selection of the action that was previously producing the effect regardless of the action mode during acquisition. To account for the differences between their own results and the results of Herwig et al. (2007), the authors proposed the different-application hypothesis (for converging evidence that the action mode can affect the application of action-effect associations, see Pfister et al., 2010; Herwig and Horstmann, 2011). According to this hypothesis, action-effect associations are acquired irrespective of the action mode, but are applied during the test phase only if an intention-based mode is adopted. Importantly, adopting an intention- or a stimulus-based mode depends not only on the current task in the test phase (free choice vs. forced choice) but also on the previous task in the acquisition phase (free choice vs. forced choice). However, the relationship between these two determining factors seem to be quite complex. According to Pfister et al. (2011) the intention-based mode is quickly adopted if participants carry out self-chosen responses (either during acquisition or test) and once adopted they will stick to this action mode even in a forced choice test phase. In contrast, participants slowly adopt a stimulus-based mode during a forced choice acquisition phase but remain in this mode only if they continue to perform forced choice actions in the test phase. Finally, Wolfensteller and Ruge (2011) suggested a third hypothesis to explain the observed effect of the action mode on ideomotor learning. In their study participants had to constantly switch between stimulus-based acquisition phases of varying lengths and forced choice test phases in which the effects were presented together with the imperative stimuli^1^. The results showed a small but reliable compatibility effect after only 12 action-effect episodes which seems to depend on contextual stability (i.e., on a consistent stimulus-response mapping). Therefore Wolfensteller and Ruge proposed the different-context hypothesis which states that action-effect associations following a stimulus-based acquisition are contextualized by means of their imperative stimuli (i.e., stimulus-action-effect episode). Such a contextualization can in principle hamper the retrieval of action-effect associations if the context (i.e., the imperative stimuli) changes between acquisition and test (cf., Godden and Baddeley, 1975).

Unfortunately, to date none of the three hypotheses, that is the different-acquisition, the different-application, and the different-context hypothesis, can satisfactorily explain all of the divergent results concerning the effect of the action mode on ideomotor learning. Thus, one main aim of the present study was to take a closer look at the emergence of action-effect associations against the background of the different-acquisition hypothesis proposed by Herwig et al. (2007).

### Action-effect binding

Up to now, we focused on the influence of the action mode on the compilation of action-effect associations that may be retrieved at least a couple of minutes after the acquisition (i.e., long-term associations or *learning*, hereafter). However, the build-up of long-term memory traces is not the only type of perceptuomotor integration that takes place when humans interact with the environment. The other type refers to a much shorter timescale and is related to one of the main characteristics of the primate brain: distributed coding (i.e., short-term integration or *binding*, hereafter)^2^. Distributed coding refers not only to features in the visual domain (e.g., shape, color, and location, see Cowey, 1985; Felleman and van Essen, 1991) and in the auditory domain (e.g., periodicity, location, and spectral shape, Brown and Wang, 2006) but also as regards the features of to-be-performed actions (e.g., direction, amplitude, and duration, Wickens et al., 1994).

Importantly, distributed coding creates numerous binding problems (Treisman, 1996), which call for some kind of integration mechanism that binds together the distributed codes belonging to the same object (e.g., color, shape, and motion of an object). Hommel (1998) argued that the binding problem holds for perceptuomotor processing as well. That is, perceptual and motor codes belonging to the same event need to be integrated, too (Hommel, 2004). Following previous work addressing the creation of “object files” (Kahneman et al., 1992), the temporarily stored outcome of this integration process was termed “event file” (Hommel, 1998).

Bindings of stimulus and action features can be assessed in the prime-probe stimulus-response task of Hommel (1998). In this paradigm each trial comprises two subtasks. In the first subtask, participants perform simple, precued left- or right key presses (R1) to the mere presence of a “Go” signal (S1) that varies randomly in form, color, and location. The effects of bindings created between S1- and R1-features on later performance are assessed in a second subtask, which is a binary-choice reaction (R2) to a pre-instructed feature (e.g., color) of a second stimulus (S2). The typical result of this type of paradigm is that performance is impaired in partial repetition trials, that is, if only the stimulus (or only the response) is repeated, compared to when both stimulus and response are repeated or when both change. This pattern of results suggests that a temporary binding of the respective codes is compiled when stimuli and actions co-occur. Repeating one feature reactivates also the associated fellow code, which, in partial repetition trials, creates a mismatch and, therefore, induces a time-consuming re-binding process (for a review, see Hommel, 2004).

Transient perceptuomotor bindings have been shown to emerge quickly (after 300 ms or less) and to remain intact for at least 4 s (Hommel and Colzato, 2004). Moreover, the temporal order of S1 and R1 does not seem to be important for perceptuomotor binding. Hommel (2005, Experiment 2) showed that stimulus features were bound to response features even if S1 follows R1 which suggests that the temporal time window for feature integration might be rather broadly defined. Thus, temporary feature binding across perception and action may take place not only in events, where the perceptual stimulus triggers the action (stimulus-based actions), but also in events, where the action triggers the perceptual event (intention-based actions). This opens up the possibility to investigate the immediate binding between actions and their effects in stimulus- and intention-based actions.

### The present study

As outlined above, Herwig and colleagues (Herwig et al., 2007; Herwig and Waszak, 2009; Herwig and Horstmann, 2011) proposed that the acquisition of action-effect associations (i.e., ideomotor learning) is affected by the action mode. The present study investigates whether the action mode also influences temporary feature bindings. Although there is already some evidence that action-effect bindings can be observed following intention-based actions (Dutzi and Hommel, 2009) and stimulus-based actions (see Hommel, 2005; Experiment 2), a direct comparison of the strength and durability of action-effect bindings following intention- and stimulus-based actions is lacking. As a consequence, it is utterly unknown whether temporary action-effect bindings, too, are affected by the action mode and one main aim of the present study was to address this gap in the literature.

We ran three experiments that compare strength as well as durability of action-effect bindings between the two action modes. Experiments 1 and 2 were designed to test how the two types of integration, that is, binding and learning, are related. Based on the different-acquisition hypothesis proposed by Herwig et al. (2007) we, see three possible relationships (see Colzato et al. (2006), for similar considerations). First, binding and learning are tightly linked (*strong dependence hypothesis*). Binding via synchronization may cause long-term modifications of synaptic efficacy as suggested by Fell et al. (2003). In this scenario, temporary bindings strengthen the association between two features mediated through Hebbian learning (i.e., neurons that fire together, wire together; Hebb, 1949), each time making the memory trace more durable. The strong dependence hypothesis assumes that the difference in ideomotor learning between intention- and stimulus-based actions, as shown by Herwig et al. (2007), is due to a difference in action-effect binding between the two modes of movement. That is, if action and effect do not wire together (ideomotor learning) in stimulus-based actions, then this might be due to the fact that action and effect features do not always fire together (temporary bindings) in the first place.

Second, ideomotor learning is completely independent of the formation of temporary action-effect bindings. Although such a *non-dependence hypothesis* is rather radical, it is not so unlikely, since binding and learning act on different time-scales and are thought to solve different problems, with bindings being involved in the problem of distributed coding and ideomotor learning being involved in the control of voluntary actions. Under this view temporary feature binding represents a representational level which is mainly used for the perception of the current event. Action-effect associations underlying ideomotor learning, however, represent a different representational level at which integrated feature assemblies are stored for the purpose of future guidance of behavior. Accordingly, there might be two crucial distinctions between both levels of representation. First, bindings as part of short-term memory depend on the actual presentation of an external effect, whereas action-effect associations as part of long-term memory depend on the internal generation of the effect. As a consequence, both representational levels might fundamentally differ in the level of detail and concreteness they are able to provide. Second, while action-effect associations underlying ideomotor learning presuppose contingent action-effect relationships (Elsner and Hommel, 2004), short-term bindings are also engaged in the perception of ever-changing action-effect relationships – just think of the different sounds one produces while talking with the mouth empty vs. full or the different ball trajectories one produces while playing pinball. Accordingly, both levels might fundamentally differ in the range of events they are able to incorporate. The non-dependence hypothesis thus assumes that the difference in ideomotor learning between intention- and stimulus-based actions (Herwig et al., 2007) do not have to be reflected in short-term bindings.

Third, binding and learning may not be as rigidly connected as assumed under the strong dependence hypothesis and not as independent as under the non-dependence hypothesis. In daily life, the particular effect that an action achieves depends tremendously on the current context. It would appear inefficient to perpetuate all episodes, that is, even those which are not needed anymore once the particular event is finished. This should especially hold true for non-contingent action-effects which cannot be reliable used for action planning. On the *weak dependence* view, binding and learning do not take place on fundamentally different levels. Instead, bindings are the building blocks for long-term associations, but only those bindings which are reliable and thus worthwhile to be preserved are further processed to form a more durable memory trace (see Colzato et al., 2006). The weak dependence hypothesis thus assumes that binding and learning are related only in case of contingency. Therefore, the difference in ideomotor learning between intention- and stimulus-based actions (Herwig et al., 2007), should only be reflected in short-term bindings of contingent action-effects, whereas it should not be reflected in short-term bindings of non-contingent action-effects.

Experiments 1 and 2 are designed to pit these three accounts against each other. The crucial difference between the experiments is that in Experiment 1 the features of the action-effect were not contingent on the action (as it is usually the case in this type of experiment), whereas in Experiments 2 they were contingent. The strong dependence hypothesis assumes that a difference in action-effect binding is the reason for the difference in ideomotor learning between intention- and stimulus-based actions. Consequently, this hypothesis predicts that intention-based actions result in both experiments in stronger binding effects than stimulus-based actions. The non-dependence hypothesis predicts that intention-based actions result neither in Experiment 1 nor in Experiment 2 in stronger binding than stimulus-based actions, since under this view learning and binding represent two different representational levels. Finally, the weak dependence hypothesis predicts that intention-based actions result only in Experiment 2 in stronger binding than stimulus-based actions, but not in Experiment 1. This is because contingent action-effects can be used only in Experiment 2 but not in Experiment 1 as building blocks for long-term associations. Experiment 3 complements Experiments 1 and 2 by directly comparing binding and ideomotor learning within one experiment.

To sum up, the present study addresses two research questions. First, are temporary bindings between action and effect features modulated by the action mode? Second, how are short-term bindings and long-term ideomotor learning related?

## Experiment 1

**Figure 1:**
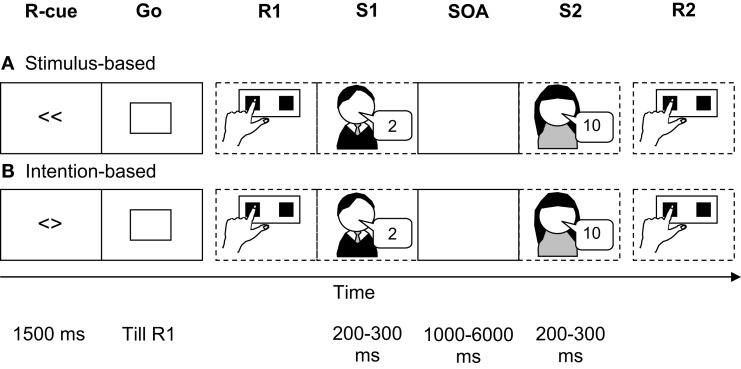
**Basic experimental setup to assess action-effect bindings**. The first subtask consists of a simple go response (R1) either in a stimulus-based **(A)** or intention-based **(B)** action mode which triggered the auditory presentation of stimulus 1 (S1). The second subtask is a binary-choice response to the number feature of stimulus 2 (S2). Note that the stimulus-onset asynchrony (SOA) was varied in Experiments 1 and 2 (1000, 2000, 6000 ms) and was constant in Experiments 3 (1000 ms).

To investigate the influence of the action mode on temporary action-effect bindings, we slightly modified the original prime-probe stimulus-response task comprising of two subtasks (see above; Hommel, 1998). In the first subtask, the first response (R1) to a neutral go signal was either freely selected (intention-based trials) or precued (stimulus-based trials). In both cases it triggered one out of four auditory effect stimuli (S1; see Figure 1). The second subtask was a speeded forced choice response (R2) to a second stimulus (S2). Moreover, we manipulated the stimulus-onset asynchrony (SOA) between S1 and S2 (1000 vs. 2000 vs. 6000 ms) to assess binding durability.

To assess the binding between features of R1 and S1, our focus was on interactions between stimulus and response repetition effects. On the basis of earlier findings regarding perceptuomotor binding (Hommel, 2005, Experiment 2), we expected that performance is impaired on partial repetition trials, in which either the response feature is repeated while the stimulus feature is alternated, or the stimulus feature is repeated while the response feature is alternated (partial repetition costs). By contrast, alternating both stimulus and response between the two subtasks of one trial should yield a performance level in the second subtask that is as good as when both are repeated. Such a pattern of results points to action-effect binding, since it implies that reactivating one feature tends to also activate the fellow feature. This, in turn, causes conflict in case of partial repetitions.

The crucial question was whether this interaction would be modulated by the action mode (intention- vs. stimulus-based). Under the strong dependence hypothesis of the relation between learning and binding, one would expect action-effect bindings to be weaker or less durable for stimulus-based than for intention-based actions. In this case the fragility of action-effect bindings in stimulus-based actions could be considered to be responsible for the effect of the action mode on ideomotor learning (see Herwig et al., 2007; Herwig and Waszak, 2009). Under the weak dependence hypothesis as well as the non-dependence hypothesis, binding should not be influenced by the action mode.

### Materials and methods

#### Participants

Sixteen adults (mean age: 24.9 years) participated. They reported having normal or corrected-to-normal vision and audition and were not familiar with the purpose of the experiment. Informed consent was obtained from all subjects.

#### Apparatus and stimuli

The experiment was controlled by a standard PC, interfaced to a 17″ monitor. The viewing distance was about 70 cm. Visual stimuli were displayed on a black background. In stimulus-based trials, two white left- or right-pointing arrows (mean extension: 0.4° × 0.7°) served as response cues and were presented in the center of the screen. In intention-based trials, the response cue was replaced by the free choice cue, i.e., two arrows pointing in different directions (<>) requesting participants to prepare a left or right key press depending on their own choice. A white rectangle (mean extension: 0.7° × 1.0°) served as a go signal for the execution of the precued/prepared response. Auditory stimuli were the English numbers “2” and “10” vocalized by a male or female voice (duration 200–300 ms). The words were presented simultaneously through the left and right speaker of a headphone. Responses were made by pressing the left or right of two keys mounted in a horizontal distance of 13.5 cm on a board with the left or right index finger.

#### Procedure and design

Each trial comprised two speeded responses. The first response (R1) was always a simple reaction to the go signal. The type of response (i.e., left or right key press) was either indicated by the response cue (stimulus-based trials) or depended on participants’ own choice (intention-based trials). R1 triggered the presentation of the first auditory effect stimulus (S1). Whether the stimulus was the number 2 or 10 vocalized by a male or a female voice was determined randomly. The second response (R2) was always a binary-choice reaction to the number feature of the second stimulus (S2). S2 was again either the number 2 or 10 vocalized by either a male or a female voice, randomly determined. Half of the participants responded to the number 2 and 10 by pressing the left and right key, respectively, whereas the other half responded according to the opposite mapping.

The sequence of events in each trial is shown in Figure 1. Following an intertrial interval of 2000 ms, a response cue or a free choice cue was presented for 1500 ms, followed by the go signal that was presented until the first response was executed. R1 triggered the presentation of S1 (50-ms onset asynchrony between R1 and S1). If R1 was not executed within 1000 ms (counted as omission) a visual warning message (too slow) was presented for 800 ms and the trial started from the beginning. If R1 was incorrect (only possible in stimulus-based trials) or anticipatory (RT < 80 ms) a visual warning message (wrong key, too fast, respectively) was presented for 800 ms and the trial continued. S2 appeared 1000, 2000, or 6000 ms after the onset of S1. Responses to S2 that were incorrect, premature (RT < 80 ms) or omitted (RT > 2000 ms) triggered presentation of the corresponding visual warning message.

The experiment was divided into four parts which were done in 1 day. Two of the four parts consisted of 3 blocks of 96 randomly ordered intention-based trials each and the remaining two parts of 3 blocks of 96 randomly ordered stimulus-based trials each. The order of the four parts was counterbalanced across participants. Participants performed 24 randomly selected practice trials at the beginning of the experiment and prior to the first switch of the action mode. That is, all in all the experiment comprised 48 practice trials and 1152 experimental trials which took approximately 4 h. Each block was composed of a factorial combination of S2 number (2 vs. 10, corresponding to left vs. right R2) and S2 gender (male vs. female), the possible relationships between S1 and S2 (repetition vs. alternation) regarding number and gender, the SOA between S1 and S2 (1000 vs. 2000 vs. 6000 ms), and the two possible relationships between R1 and R2 (repetition vs. alternation). In intention-based blocks, in contrast, the relationship between R1 and R2 could not be determined *a priori* because R1 depends on participants’ free choice. In these blocks participants were instructed to use the left and right key for the first response about equally often and in a random order. Participants could take a break after each block.

### Results

For the sake of clarity and according to our main question (i.e., action-effect bindings for intention- and stimulus-based actions), we present only the results of subtask 2 and, specifically only the reliable effects in the main text. The Appendix presents the results of subtask 1 as well as two tables which provide a detailed overview of the means (see Table A1 in Appendix) and ANOVA outcomes (see Table A2 in Appendix) for RTs and error rates obtained for subtask 2. After excluding trials in which R2 was anticipated or omitted (0.2%), R2 data were analyzed as a function of the action mode (intention- vs. stimulus-based), SOA (1000 vs. 2000 vs. 6000 ms), and repetition vs. alternation of stimulus number, gender, and response. Analyses of variance (ANOVA) with the factors Action mode (intention- vs. stimulus-based), Response (repetition vs. alternation), Number (repetition vs. alternation), Gender (repetition vs. alternation), and SOA (1000 vs. 2000 vs. 6000 ms) were performed on error rates and error-free RTs by using a five-way design for repeated measures. Violations of sphericity were corrected using the Huynh–Feldt ε. The significance criterion was set to *p* < 0.05 for all analyses.

#### Reaction times

The RT analysis yielded five reliable effects and importantly, none of these effects interacted with the action mode (*p*s > 0.24). There were main effects of SOA, response, and gender. These main effects indicated faster responses with increasing SOA (661, 637, and 603 ms for SOA of 1000, 2000, and 6000 ms, respectively), for response alternations (643 and 624 ms for response repetitions and alternations, respectively), and for gender repetitions (623 and 644 ms for gender repetitions and alternations, respectively). The main effect of gender was further modified by an interaction with number, indicating an integration of the auditory stimulus features number and gender.

**Figure 2:**
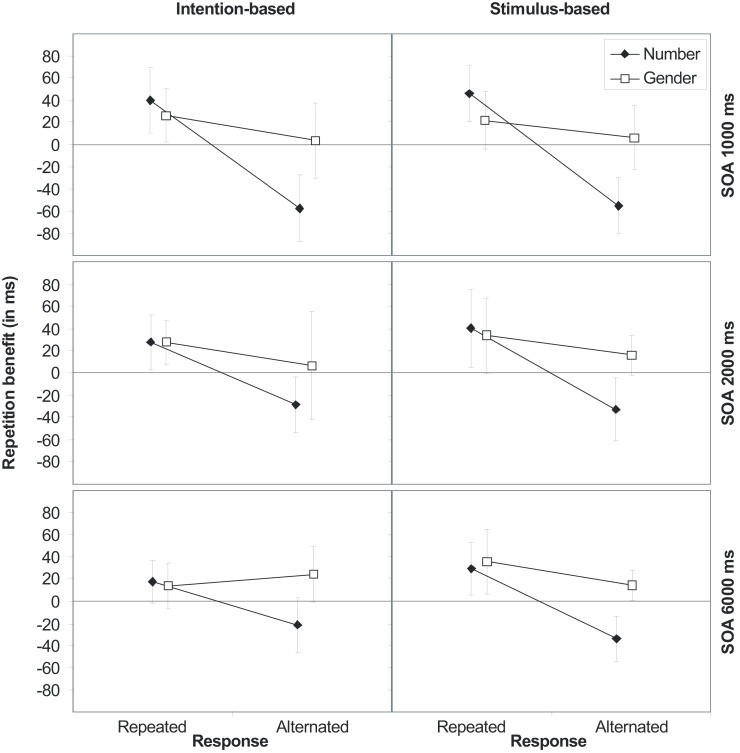
**Stimulus (S1–S2) repetition benefits (RT_alternation_-RT_repetition_) in Experiment 1 for stimulus features number and gender for intention-based (left panels) and stimulus-based trials (right panels) as a function of response relation (R1–R2 repetition or alternation) and stimulus-onset asynchrony (SOA 1000, 2000, and 6000 ms, from top to bottom)**. Error bars represent the 95% confidence interval of individual means. If error bars do not cross the midline, the repetition benefit (or cost) is significantly different from zero (*p* < 0.05).

More importantly, the main effect of response was modified by an interaction with number, indicating action-effect binding. Figure 2 shows the relative repetition benefit for each stimulus dimension (i.e., the mean RT difference between number/gender alternation and number/gender repetition; note that the values depicted in Figure 2 are differences of averaged values given in Table A1 in Appendix) as a function of the relationship between R1 and R2 separated for intention- and stimulus-based trials and the three SOAs. A positive difference indicates that participants responded faster for stimulus repetitions than alternations, whereas a negative difference indicates faster reactions for stimulus alternations than repetitions. As Figure 2 clearly shows, repeating stimulus number produces a benefit if, and only if, the response is also repeated. If the response is alternated, the repetition benefit turns into an alternation benefit. This was true for all three SOAs.

#### Error rates

The error rates overall mirrored the RTs but produced some additional effects. Importantly, once again none of the reported effects was modified by the action mode (*p*s > 0.27). As concerns the main effects, participants committed fewer errors with increasing SOA and response alternations. However, in contrast to the RT data, participants committed fewer errors with gender alternations (3.0 and 2.5% for gender repetitions and alternations, respectively). Thus a speed-accuracy trade-off can be excluded for the factors SOA and response, but not for gender. The main effect of response was modified by an interaction with SOA, indicating an increased alternation benefit with the medium SOA of 2000 ms.

Of importance, response interacted with number as well as with gender, indicating that each stimulus dimension was separately integrated with the response. Repeating both the number and the response or alternating both (1.8 and 0.9%, respectively) decreased the error rate, whereas the error rate increased if only one, but not the other, was repeated (number repeated: 3.5%; response repeated: 4.9%). Likewise, a response repetition was easier if gender was also repeated than alternated (3.2 and 3.4%, respectively), whereas a response alternation was easier if gender was also alternated than repeated (1.7 and 2.8%, respectively). Moreover, action-effect bindings for both effect features interacted with SOA. Separate ANOVAs for each SOA showed both interactions to be significant only for the SOAs of 1000 [response × number: *F*(1,15) = 36,80, *p* < 0.001, $\eta_{p}^{2}=0.71,$ response × gender: *F*(1,15) = 12.06, *p* = 0.003, $\eta_{p}^{2}=0.45$] and 2000 ms [response × number: *F*(1,15) = 24.30, *p* < 0.001, $\eta_{p}^{2}=0.62;$ response × gender: *F*(1,15) = 4.83, *p* = 0.044, $\eta_{p}^{2}=0.24$] but not for the SOA of 6000 ms (*p*s > 0.143).

### Discussion

As shown in Figure 2, the effect of stimulus repetition was clearly dependent on whether or not the response was also repeated. Thus, Experiment 1 suggests that the co-occurrence of action and auditory codes triggered by the action results in the temporary binding between the involved perceptual and motor features. Comparable to studies investigating perceptuomotor binding (e.g., Hommel, 1998), action-effect bindings were pronounced for the task relevant stimulus feature (i.e., number). Moreover, the analysis of RTs of Experiment 1 showed action-effect bindings to remain intact for at least six seconds – a finding that extends the results regarding the durability of perceptuomotor bindings by 2 s (Hommel and Colzato, 2004).

More importantly, Experiment 1 did not show any influence of the action mode on the strengths or durability of the action-effect bindings. That is, short-term action-effect bindings were comparably strong and durable following intention- and stimulus-based actions. This observation is in contrast to the predictions derived from the strong dependence hypothesis of binding and learning. Thus, the finding of Herwig et al., 2007; see also Herwig and Waszak, 2009; Herwig and Horstmann, 2011) that ideomotor learning is affected by the action mode does not seem to be due to an elementary difference in action-effect binding.

However, the dissociation of the effect of the action mode on binding and learning is in accord with the non-dependence as well as the weak dependence hypothesis. If binding and learning actually represent two independent representational levels (as suggested by the non-dependence hypothesis), one would not expect binding and learning to be influenced by the same factors. According to the weak dependence hypothesis action-effect binding is a necessary, but not a sufficient precursor for long-term ideomotor learning. In this scenario, the action mode might determine whether or not the repeated formation of identical transient bindings forms a memory trace. Metaphorically speaking, bindings may be regarded as building blocks that are constructed whenever an effect is produced in close temporal contiguity by an action regardless of whether the action was externally or internally selected. However, only intention-based actions, but not stimulus-based actions, may provide the glue necessary to agglutinate these building blocks to form a durable memory trace.

This notion can only be tested if one effect feature is produced contingently by one and not the other action. In Experiments 1 each effect feature was produced by each action with the same probability. Consequently, distinct action-effect relations could not be established. Therefore, we implemented contingent action-effect mappings in Experiments 2 and 3.

## Experiment 2

As pointed out above, one reason for the missing influence of the action mode on the formation and durability of action-effect bindings might be related to the fact that each effect feature was produced by each action with the same probability. It is possible that due to this missing contingency between action and effect features binding and learning are unrelated as suggested by the weak dependence hypothesis. To address this issue, Experiment 2 was conducted, in which each action (R1) contingently produced one specification of the irrelevant effect feature of S1 (i.e., gender). For example, pressing the left key led to the auditory presentation of the number “2” or “10” spoken by a female voice, whereas pressing the right key resulted in the presentation of the number “2” or “10” spoken by a male voice.

Such a contingency manipulation should in principle enhance ideomotor learning (Elsner and Hommel, 2004). Importantly, if the weak dependence hypothesis holds (i.e., if binding and learning are only related in case of a contingent action-effect relationships), this enhancement should be reflected in partial repetition costs as well. This is because in Experiment 2 R2 may be affected by two factors: the *event file* compiled during the fist subtask of each trial and the *memory trace* emerging through the repeated experience of the contingent action-effect mapping. Both factors should entail RT costs if only the gender or the response is repeated while the other feature is alternated (i.e., partial repetition costs). Consider, for instance, an action-effect mapping for R1–S1 that links a left key press with a female voice (*F*) and a right key press with a male voice (*M*). Moreover, the stimulus-response mapping rule for S2–R2 be to respond to the number two (*2*) and ten (*10*) by pressing the right and left key, respectively. If S2 is the number two spoken by a female voice (*2F*), this might lead to a conflict in initiating R2 because female may automatically activate the left response due to the compiled memory trace, whereas 2 calls for a right response due to the instructed mapping. Likewise, if S2 is *10M*, 10 calls for a left whereas male calls for a right response. In contrast, no conflict arises if S2 is *2M* or *10F*, because the number as well as the gender feature call for the same response. Importantly, in the given example, *2F* and *10M* would also be the partial repetitions with respect to R1–S1, because a left R1 always triggers S1 spoken with a female voice and a right R1 always triggers S1 spoken with a male voice (leaving *2M* and *10F* as complete repetitions or complete alternations). Accordingly, if contingency determines whether binding and learning are related or not, one would expect R2 to be influenced by the previously compiled event file and the accumulating memory trace only for intention-based actions. In contrast, for stimulus-based actions R2 should be affected solely by the event file, resulting in a three-way interaction of response, gender, and action mode.

### Materials and methods

Participants were 32 adults (mean age 24.2 years) who fulfilled the same criteria as those in Experiments 1. The method was the same as in Experiment 1, with the following exceptions. The gender feature of S1 depended on R1 (e.g., left key press/female voice, right key press/male voice), whereas there was, as in Experiment 1, no contingency regarding the number feature of S1. The action-effect mapping was counterbalanced across participants. Participants were not informed about the contingency manipulation^3^. Moreover, in contrast to Experiment 1, the action mode (intention- vs. stimulus-based) was manipulated between subjects to avoid transfer effects (i.e., to be sure that R2 following stimulus-based actions was not influenced by accumulated memory traces that were established following intention-based actions). The experiment consisted of 6 blocks of 96 randomly ordered trials and took approximately 2 h. Each block was composed of the possible combinations of two R1 alternatives (left vs. right), two S1 alternatives (2 vs. 10, either male or female voice depending on R1), four S2 alternatives (2-male, 2-female, 10-male, 10-female), three SOAs (1000, 2000, 6000 ms), and two repetitions of each combination. Importantly, the independence of repetition vs. alternation regarding number, gender, and response remained unchanged by the contingency manipulation, since R2 was always a reaction to the number feature (and not to the gender feature) of S2.

### Results

As for Experiment 1, we present only the reliable effects of subtask 2 in the main text. The Appendix presents the results of subtask 1 as well as the means (see Table A3 in Appendix) and ANOVA outcomes (see Table A4 in Appendix) for RTs and error rates obtained for subtask 2. After excluding trials in which R2 was anticipated or omitted (0.3%), R2 data were analyzed as in Experiment 1. ANOVA with the between subjects factor Action mode (intention- vs. stimulus-based) and the within subjects factors Response (repetition vs. alternation), Number (repetition vs. alternation), Gender (repetition vs. alternation), and SOA (1000 vs. 2000 vs. 6000 ms) were performed on error rates and error-free RTs.

#### Reaction times

The RT analysis produced various reliable effects. Importantly, action-effect bindings were not modulated by the action mode (*p*s > 0.24). Beside the main effects of SOA, response, number, and gender, indicating faster responses with increasing SOA (586, 566, and 544 ms), faster responses for response alternations (575 and 556 ms), number repetitions (560 and 571 ms), and gender repetitions (560 and 571 ms), all three two-way interactions between response, number, and gender reached significance, indicating stimulus feature as well as action-effect bindings.

**Figure 3:**
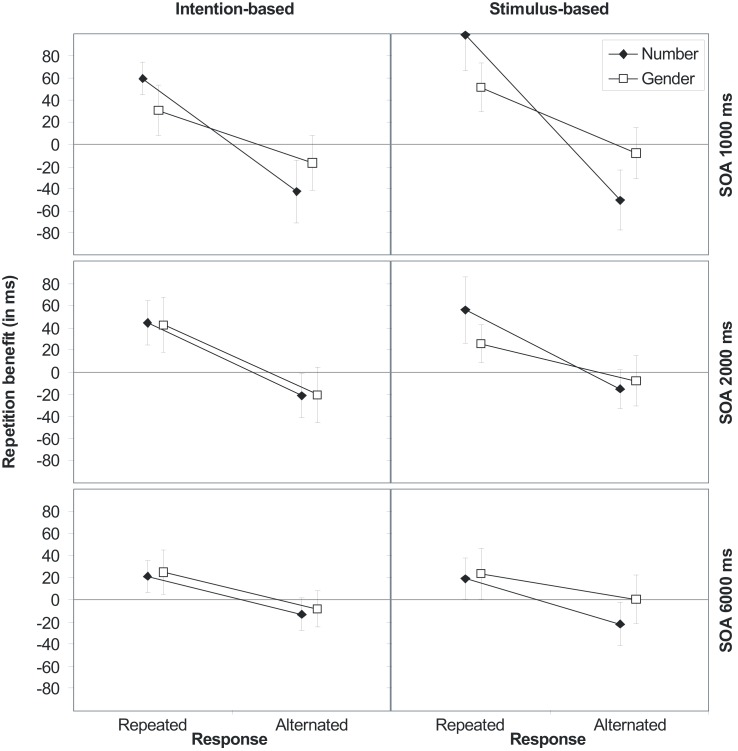
**Stimulus (S1–S2) repetition benefits (RT_alternation_-RT_repetition_) in Experiment 2 for stimulus features number and gender for intention-based (left panels) and stimulus-based group (right panels) as a function of response relation (R1–R2 repetition or alternation) and stimulus-onset asynchrony (SOA 1000, 2000, and 6000 ms, from top to bottom)**. Error bars represent the 95% confidence interval of individual means. If error bars do not cross the midline, the repetition benefit (or cost) is significantly different from zero (*p* < 0.05).

As shown in Figure 3, repeating stimulus number or gender produce a benefit if, and only if, the response is also repeated, whereas the repetition benefit turns into an alternation benefit if the response is alternated. Although the response × number interaction was further modified by SOA, separate ANOVAs showed the interaction to be significant for all three SOAs. Noteworthy, there was a three-way interaction of response × number × gender that was due to a decrease of the response-by-gender interaction-effect if the number was alternated. However, separate ANOVAs showed the response-by-gender interaction to be significant for number repetitions [(*F*(1,30) = 40.91, *p* < 0.001, $\eta_{p}^{2}=0.58$] as well as number alternations [*F*(1,30) = 4.63, *p* = 0.040, $\eta_{p}^{2}=0.13$].

#### Error rates

The error rates overall mirrored the RTs. Importantly, once again none of the reported effects was modified by the action mode. Beside the main effects of SOA and response, the two binary interactions between response and number and response and gender followed the same pattern as the RTs and indicated action-effect binding. That is, repeating both the number and the response or alternating both (2.2 and 0.7%, respectively) decreased the error rate, whereas the error rate increased if only one, but not the other, was repeated (number repeated: 4.4%; response repeated: 4.8%). In the same way, repeating both the gender and the response or alternating both (3.1 and 1.7%, respectively) decreased the error rate, whereas the error rate increased if only one, but not the other, was repeated (gender repeated: 3.4%; response repeated: 4.0%). Both action-effect bindings were further modified by SOA. Separate ANOVAs showed the response-by-number interaction to be significant for all SOAs, whereas the response-by-gender interaction was only significant for the SOAs of 1000 and 2000 ms but not for the SOA of 6000 ms (*p* > 0.37). The three-way interaction of response × number × gender followed the same pattern as the RTs and was due to a decrease of the response-by-gender interaction-effect if the number was alternated.

### Discussion

First of all, Experiment 2 yielded a reliable response-by-gender interaction, i.e., an interaction between the response and the contingent (first subtask), but task irrelevant (second subtask) effect feature. Although pronounced for repetitions of the task relevant feature this action-effect binding occurred also for alternations of the task relevant feature. This pattern of results suggests two things. First, each effect feature (i.e., the relevant but non-contingent number feature as well as the irrelevant but contingent gender feature) is separately bound to the action. Second, in addition to these single feature bindings, there is also a binding between the action and a compound of both effect features.

More importantly, the action mode did not modify the bindings’ strength or durability even under action-effect contingency. We also reran the ANOVA on RTs with the additional factor half of the experiment (first half vs. second half) to test whether the action mode modifies bindings only after some experience with the contingent action-effect mapping. This ANOVA also did not provide any evidence for an effect of the action mode (four-way interaction of Experiment half, Response, Gender, and Action mode, *p* > 0.78).

To sum up, the outcome of Experiment 2 failed to find an influence of the action mode on bindings between actions and their ensuing effect features, even though action and effect (gender) were contingent across the experiment. In light of previous studies showing that ideomotor learning can be affected by the action mode (Herwig et al., 2007), this finding is more in line with the non-dependence hypothesis than with the predictions derived from the weak dependence hypothesis of binding and learning. This is because the latter hypothesis assumes that binding and learning are related under action-effect contingency and thus should be influenced by the same factors.

However, two caveats impinging on the present data has to be taken into account before these results can be taken as evidence that ideomotor learning is more ore less independent of short-term action-effect bindings. First, intermingling effect-producing actions (subtask one) with choice responses to stimuli (subtask two) in Experiment 2 might have interfered with ideomotor learning. Second, up to now, we investigated binding (current study) and learning (Herwig et al., 2007; Herwig and Waszak, 2009; Herwig and Horstmann, 2011) in different experiments using different experimental designs.

## Experiment 3

Experiment 3 was conducted to deal with these two caveats. To this end, we assessed both, bindings during an acquisition phase and ideomotor learning in a subsequent test phase. The acquisition phase was modeled after Experiment 2 so that once again the gender feature of S1 depended on R1. (We call the first part of the experiment acquisition phase, because in participants should acquire long-term memory traces. However, at the same time the acquisition phase served to test for temporary bindings, just as in Experiments 1 and 2.) In the additional test phase, participants were instructed to respond to the number feature of new stimuli (the English numbers “four” or “five”) either with a left or a right key press. Importantly, the new stimuli were spoken either with a male or a female voice. If ideomotor learning occurs, than one would expect to find facilitation if the instructed stimulus feature number calls for the same response to which the task irrelevant feature gender is associated (compatible trials). In contrast, interference should occur if number and gender call for different responses (incompatible trials).

### Materials and methods

Thirty-two adults (mean age 25.1 years) who fulfilled the same criteria as those in the previous experiments participated in this single session experiment of about 1 h. The experiment was divided in an acquisition and a test phase. The method used during acquisition was the same as in Experiment 2, with the only exception that there was no SOA manipulation and S2 always appeared 1000 ms after the onset of S1. The acquisition phase consisted of 2 blocks of 96 randomly ordered trials. Half of the participants executed R1 in an intention-based way, whereas the other half executed R1 in a stimulus-based way. After completing the acquisition phase, participants received an on-screen instruction of the required stimulus-response mapping for the test phase. In each test trial one out of four possible new stimuli (“four” or “five” vocalized by a male or a female voice) was presented. Half of the participants were instructed to respond to the number “four” with a left key press and to the number “five” with a right key press, whereas this mapping was reversed for the other half of participants. The next trial started 2000 ms after the response. The test phase comprised 200 randomly ordered trials (100 compatible and 100 incompatible trials).

### Results

#### Acquisition phase

The Appendix presents the results of subtask 1 as well as the means (see Table A5 in Appendix) obtained for subtask 2. After excluding trials in which R2 was anticipated or omitted (0.1%), an ANOVA on R2 data was performed on error rates and error-free RTs with the between subjects factor Action mode (intention-based vs. stimulus-based) and the within subjects factors Response (repetition vs. alternation), Number (repetition vs. alternation), and Gender (repetition vs. alternation).

**Figure 4:**
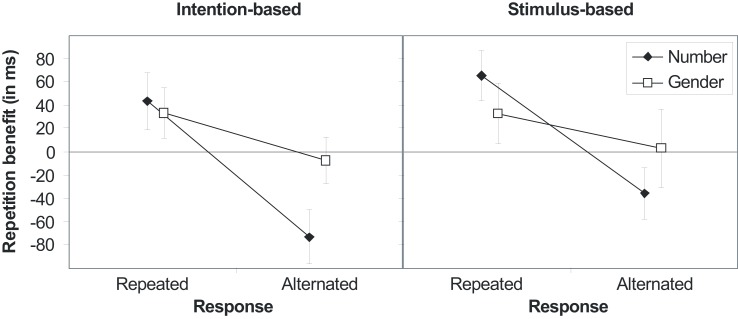
**Stimulus (S1–S2) repetition benefits (RT_alternation_-RT_repetition_) in Experiment 3 for stimulus features number and gender for intention-based (left panel) and stimulus-based group (right panel) as a function of response relation (R1–R2 repetition or alternation)**. Error bars represent the 95% confidence interval of individual means. If error bars do not cross the midline, the repetition benefit (or cost) is significantly different from zero (*p* < 0.05).

The RTs produced six reliable effects. Once again, action-effect bindings were not modified by the action mode (*F*s < 1, *p*s > 0.441). Beside the main effect of gender [*F*(1,30) = 7.80, *p* = 0.009, $\eta_{p}^{2}=0.21$], indicating faster response if gender was repeated than alternated (496 and 511 ms, respectively), there was an interaction of number with action mode [*F*(1,30) = 5.67, *p* = 0.024, $\eta_{p}^{2}=0.16$]. This interaction was due to faster responses if number was repeated in the stimulus-based group (477 and 491 ms), whereas responses were slower if number was repeated in the intention-based group (530 and 516 ms). Of importance, all three binary interactions between response, number, and gender reached statistically significance. That is the interaction of number and gender [*F*(1,30) = 41.32, *p* < 0.001 $\eta_{p}^{2}=0.58$] indicated stimulus feature binding, whereas the interactions of response and number [*F*(1,30) = 104.67, *p* < 0.001, $\eta_{p}^{2}=0.78$] and response and gender [*F*(1,30) = 5.68, *p* = 0.024, $\eta_{p}^{2}=0.16$] indicated action-effect bindings. As depicted in Figure 4, repeating stimulus number or gender produce a benefit only if the response is repeated, whereas this benefit turns into in alternation benefit (as concerns the number feature) or vanishes (as concerns the gender feature) if the response is alternated. The sixth reliable effect was a three-way interaction of response × number × gender [*F*(1,30) = 4.47, *p* = 0.043, $\eta_{p}^{2}=0.13$]. Separate ANOVAs revealed that the response-by-gender interaction was pronounced for complete repetitions and alternations of response and number (*p* < 0.001), whereas it was absent for partial repetitions (*p* = 0.462).

The error rates mirrored both action-effect bindings observed in the RTs. Importantly, action-effect bindings were not modified by the action mode. The response-by-number interaction [*F*(1,30) = 17.42, *p* < 0.001, $\eta_{p}^{2}=0.37$] as well as the response-by-gender interaction [*F*(1,30) = 7.66, *p* = 0.010, $\eta_{p}^{2}=0.20$] reached statistical significance. Repeating both the number and the response or alternating both (2.5 and 1.1%, respectively) decreased the error rate, whereas the error rate increased if only one, but not the other, was repeated (number repeated: 5.4%; response repeated: 6.1%). Likewise, complete repetitions or alternations of response and gender decreased the error rate (3.1 and 2.4%, respectively), whereas the error rate increased with partial repetitions (gender repeated: 4.0%; response repeated: 5.5%). The ANOVA of error rates yielded no further reliable effects.

#### Test phase

**Figure 5:**
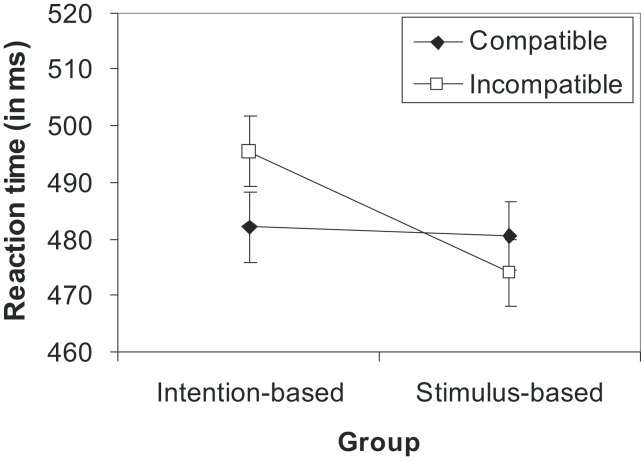
**Mean reaction times in the test phase of Experiment 3 as a function of group and compatibility**. Error bars represent within-subject standard errors, calculated separately for each group (Loftus and Masson, 1994).

Error rates and error-free mean RTs of the test phase were analyzed by mixed ANOVAs as a function of the action mode during the acquisition phase (between subjects factor) and compatibility (within-subject factor). The analysis of RTs yielded a significant interaction of the action mode and compatibility [*F*(1,30) = 5.41, *p* = 0.027, $\eta_{p}^{2}=0.15$]. None of the main effects reached statistical significance (*F*s < 1, *p* < 0.430). As shown in Figure 5 and as revealed by separate *t*-test, participants responded significantly faster on compatible (482 ms) than incompatible trials (495 ms) in the intention-based group [*t*(15) = −2.16, *p* = 0.047, *d* = 0.54, two-tailed], whereas there was no compatibility effect for the stimulus-based group [*t*(15) = 1.10, *p* = 0.287, *d* = 0.28, two-tailed]. The ANOVA on error rates did not yield any reliable effect. Suffice it to say that errors did not counteract the RT data, and thus, a speed-accuracy trade-off can be excluded.

### Discussion

Experiment 3 perfectly replicated the finding that the action mode affects binding and learning differently: action-effect bindings were unaffected by the action mode (replicating Experiments 1 and 2), whereas ideomotor learning was observed for intention-based actions only (replicating Herwig et al., 2007; Herwig and Waszak, 2009; Herwig and Horstmann, 2011). However, there are some points that need to be mentioned. First, unlike previous findings the compatibility effect for the intention-based group in Experiment 3 was rather small (13 ms instead of 30–70 ms). This might be due to the fact that in the present study the contingent effect feature was either task irrelevant in the test phase or had to compete against another task relevant effect feature during acquisition (or even a combination of both). Second, the interaction-effect seems to be driven more by interference than facilitation in the intention-based group as indicated by elevated reaction times to incompatible effects but comparable reaction times to compatible effects. Moreover, comparable to Experiment 2 action-effect bindings for gender were pronounced for complete repetitions and alternations of response and number, whereas, contrary to Experiment 2, they were not reliable for partial repetitions. This may indicate that there was a binding between the action and a compound of both effect features only. If correct, this interpretation would cast doubt on the notion that the results unequivocally support the non-dependence hypothesis. This is because we did not test for ideomotor learning of the action-compound association. However, Experiment 2 revealed that participants actually bind response and gender features even for number alterations. Thus, the difference between both experiments might be rather due to differences in power (576 vs. 200 trials in Experiment 2 and 3, respectively) than to qualitative differences underlying action-effect binding. To sum up, different effects of the action mode on short-term bindings and ideomotor learning were replicated within one experiment which rule out that intermingling effect-producing actions (subtask one) with choice responses to stimuli (subtask two) in Experiment 2 might have interfered with ideomotor learning.

## General Discussion

The present study aimed at addressing two research questions: are temporary bindings between action and effect features modulated by the action mode? How are temporary bindings and long-term ideomotor learning related?

Concerning the first research question, all experiments reported above showed strong and long-lasting (up to 6 s) action-effect bindings, not only for intention-based but also for stimulus-based actions. Importantly, we found no indication for the strength and durability of these bindings being dependent on the action mode. Hence, in contrast to ideomotor learning, temporary action-effect bindings are not modulated by the action mode. This finding is corroborated by the results of Janczyk et al. (2012). They used a different approach to assess the strength (but not durability) of short-term bindings following intention- and stimulus-based actions which required free- instead of forced choice responses in subtask 2 (Dutzi and Hommel, 2009). Accordingly, repetition rates were analyzed instead of RTs and error rates. With this slightly different experimental approach, Janczyk and colleagues also found immediate action-effect bindings for both types of actions. Interestingly, stimulus-based actions in their study even increased the bias to repeat the response if the stimulus was also repeated. However, they also pointed out that this observation does not necessarily imply stronger action-effect bindings for stimulus-based action.

Concerning our second research question about the relationship of binding and learning, the results of the present study are in line with the non-dependence hypothesis and suggest that binding and learning take place at different representational levels. Moreover, the results are in line with the study from Colzato et al. (2006). These authors manipulated the conjunction learning strength of a particular shape-color conjunction (i.e., their study dealt with bindings between stimulus features). They observed that bindings were not affected by previous learning (hence, Colzato et al., 2006 investigated the impact of learning on binding, whereas the present study focused on the impact of binding processes on learning). Colzato and colleagues concluded that learning is not a direct consequence of temporary bindings accumulating through Hebbian learning (i.e., neurons that fire together, wire together; Hebb, 1949).

It seems, thus, that different neural mechanisms mediate binding and learning. It has been proposed that temporary feature binding can be accomplished by selective synchronization of the firing patterns of feature coding neurons (e.g., von der Malsburg, 1999; Engel and Singer, 2001). Because temporal synchronization avoids combinatorial problems that are inherent in the principle of higher-order representations (e.g., cardinal cells, Barlow, 1972), it is well suited to flexibly represent a large number of frequently changing feature combinations. In contrast, learning seems to rely on strengthening of connections between neuron populations via long-term potentiation (e.g., Zalutsky and Nicoll, 1990; Bliss and Collingridge, 1993). Consequently, there has to be an additional process of consolidation that transforms transient bindings into durable memory traces (for discussions of consolidation processes in motor learning, see McGaugh, 2000; Robertson et al., 2004; Hotermans et al., 2006) since otherwise the brain is left without a trace when synchronization is gone (Wagner, 2001).

This brings us back to the question of why the action mode can affect the detection of action-effect associations that may be retrieved at least a couple of minutes after the acquisition. From a functional perspective, it seems to be crucial that consolidation is selective, i.e., irrelevant aspects of what binding processes integrate have to be filtered out while relevant aspects that might be needed on a later occasion have to be transformed into durable memory traces. Such filtering might be achieved by redundancy compression which is proposed to be regulated by the hippocampus (Gluck and Myers, 1993; Gluck et al., 2003). Redundancy compression assures that coincidental context information will come to be represented by a decreasing amount of neurons, whereas the relevant elements of the task remain well represented. In case of stimulus-based actions, the relevant element of the task is the stimulus-response rule specifying which motor routines action-relevant stimuli habitually require. In case of intention-based actions, in contrast, it is the action-effect rule that is used to control behavior (Pfister et al., 2010; Herwig and Horstmann, 2011). Accordingly, differences in ideomotor learning might be due to the (redundant) effect stimuli being compressed in stimulus-based actions but not in intention-based actions. A crucial question that needs to be addressed is whether short-term bindings are actually immune to the proposed filtering operations accompanying learning. Although to date there is no direct evidence validating or refuting this claim in the domain of action-effect learning and binding, there are already some tentative hints. These hints can be drawn from studies investigating stimulus-outcome learning and stimulus-response binding. For instance, Kruschke and Blair (2000) suggested that the learning phenomenon of conditioned blocking is driven by shifting attention away from the redundant information. Blocking refers to a situation in which stimulus-outcome learning is apparently reduced for a new cue accompanying an old cue that was already learned to perfectly predict an outcome. On the contrary, bindings seem to be not much affected by attention (Hommel, 2005) which suggests that bindings might be immune to redundancy compression. This claim would also fit to our observation, that even in the second half of Experiment 2 (i.e., when redundancy compression was probably at work) the action mode did not modify binding strength or durability.

With the refinement presented above the different-acquisition hypothesis put forward by Herwig et al. (2007) is also capable of explaining the divergent results of Wolfensteller and Ruge (2011) who observed action-effect learning following only 12 stimulus-based actions-effect episodes. Given their short acquisition phases it is not unlikely that during the test phase, the effect stimuli were not yet fully compressed leading to a small but reliable compatibility effect. However, based on the present data we cannot determine whether the action mode affects the acquisition of long-term action-effect associations prior to their retrieval (as suggested by the different-acquisition hypothesis of Herwig et al., 2007) or whether the action mode solely affects the application of these associations during the test phase (as suggested by the different-application hypothesis of Pfister et al., 2011). A satisfactory answer to this question will require future research directly testing whether intricate differences in the time course of adopting and switching action modes, as assumed by the different-application hypothesis, are actually responsible for the divergent results of Herwig et al. (2007) and Pfister et al. (2011). Obviously, the same holds true for alternative assumptions attributing the divergent results for instance to differences in the detection sensitivity of free choice and forced choice test phases which might be responsible for the detection or non-detection of weak action-effect associations following a stimulus-based acquisition.

In summary, the present experiments clearly show that transient action-effect bindings are unaffected by the action mode. At the same time, durable memory traces linking actions and their effects were detected only following intention-based but not stimulus-based actions. As a consequence the effect of the action mode on ideomotor learning cannot merely be a result of accumulated action-effect bindings. Instead, we suggest that only those episodic bindings are selectively perpetuated and retrieved that integrate action-relevant aspects of the processing event, that is, in case of intention-based actions, the link between action and ensuing effect.

## Conflict of Interest Statement

The authors declare that the research was conducted in the absence of any commercial or financial relationships that could be construed as a potential conflict of interest.

## Footnotes

^1^Contrary to previous studies (e.g., Elsner and Hommel, 2001; Herwig et al., 2007; Pfister et al., 2011), Wolfensteller and Ruge (2011) made their participants switch repeatedly between acquisition and test phases in one experimental session. It is possible that this procedure has replaced incidental ideomotor learning by intentional learning since participants might have noticed that the effects will be relevant in the following test phases.

^2^It should not go unnoticed that the divide between learning and binding is, at the same time, a divide between short-term and long-term memory. The question how binding and learning are related is thus also a question about how short-term memory representations are consolidated and translated into long-term memory. We will come back to this question in the General Discussion.

^3^A post-test survey revealed that only 5 (all in the intention-based group) of the 32 participants recognized the correct action-effect mapping among four alternatives. The four alternatives they had to choose from were (1) left keypress = male voice; (2) left keypress = female voice; 3 = right keypress = male voice; 4 = right keypress = female voice.

## Appendix

### Experiment 1

#### Subtask 1

The first response (R1) to the onset of the go signal was correctly carried out in stimulus-based trials in 330 ms, on average, and in intention-based trials in 375 ms, on average [*t*(15) = 3.49, *p* = 0.003, *d* = 0.87, two-tailed]. Errors of R1 in stimulus-based trials were rare (0.2%), as were anticipations and response omissions (stimulus-based: 0.4 and 1.4%, respectively; intention-based: 0.5 and 1.3%, respectively). The distribution of left-hand vs. right-hand key presses in intention-based trials was nearly equal (49.1 vs. 50.9%; average of absolute difference between left- and right-hand key presses = 5.9%) and provided a comparable amount of response repetitions and alternations (50.7 vs. 49.3%).

#### Subtask 2

**Table A1:** **Means of mean reaction times (RTs, in ms) and error rates (ER) for R2 in Experiment 1 as a function of the relationship between S1 and S2 and between R1 and R2 and stimulus-onset asynchrony for intention-based and stimulus-based trials**.

Stimulus feature repeated	Response							
	Intention-based				Stimulus-based			
	Repeated		Alternated		Repeated		Alternated	
	RT	ER	RT	ER	RT	ER	RT	ER
**SOA 1000 ms**								
Neither	673	8.8	607	0.5	701	5.0	630	0.8
Number	669	1.0	679	3.8	683	1.1	706	3.5
Gender	684	6.5	613	0.8	708	5.8	641	0.8
Both	601	0.5	664	6.6	630	1.3	684	9.2
**SOA 2000 ms**								
Neither	658	4.8	608	0.6	673	5.3	615	0.3
Number	657	2.7	646	1.3	657	4.0	668	2.4
Gender	657	6.0	608	1.6	663	4.7	615	0.5
Both	598	2.4	635	4.8	593	0.8	636	4.5
**SOA 6000 ms**								
Neither	624	1.4	577	1.6	658	3.2	581	1.0
Number	602	2.4	615	1.9	629	1.3	630	2.1
Gender	607	3.8	564	0.8	622	3.0	578	1.3
Both	589	2.5	574	1.5	589	1.6	604	0.5

*SOA, stimulus-onset asynchrony*.

**Table A2:** **Results of analysis of variance on mean reaction times of correct responses (RT) and error rates (ER) for Experiment 1**.

Effect	*df*	RT_R2_			ER_R2_		
		*F*	$\eta_{p}^{2}$	*p*	*F*	$\eta_{p}^{2}$	*p*
SOA	2,30	8.89	0.37	**	8.95	0.37	**
Am	1,15	0.46	0.03		0.64	0.04	
Res	1,15	11.09	0.43	**	8.42	0.36	*
Num	1,15	0.72	0.05		0.60	0.04	
Gen	1,15	15.23	0.50	**	4.54	0.23	*
SOA × Am	2,30	1.09	0.07		0.02	0.00	
SOA × Res	2,30	0.50	0.03		3.43	0.19	*
SOA × Num	2,30	1.06	0.07		0.07	0.00	
SOA × Gen	2,30	0.49	0.03		1.21	0.07	
Am × Res	1,15	0.03	0.00		0.79	0.05	
Am × Num	1,15	0.06	0.00		2.04	0.12	
Am × Gen	1,15	0.21	0.01		0.37	0.02	
Res × Num	1,15	46.54	0.76	**	35.18	0.70	**
Res × Gen	1,15	3.17	0.17		5.81	0.28	*
Num × Gen	1,15	16.66	0.53	**	2.13	0.12	
SOA × Am × Res	2,30	0.10	0.01		0.96	0.06	
SOA × Am × Num	2,30	0.11	0.01		3.21	0.12	
SOA × Am × Gen	2,30	0.20	0.01		3.97	0.21	*
SOA × Res × Num	2,30	2.96	0.17		22.11	0.60	**
SOA × Res × Gen	2,30	0.50	0.03		6.17	0.29	**
SOA × Num × Gen	2,30	3.24	0.18		4.12	0.22	*
Am × Res × Num	1,15	1.37	0.08		0.00	0.00	
Am × Res × Gen	1,15	0.30	0.02		0.68	0.04	
Am × Num × Gen	1,15	0.04	0.00		0.02	0.00	
Res × Num × Gen	1,15	1.01	0.06		7.14	0.32	*
SOA × Am × Res × Num	2,30	0.34	0.02		1.34	0.08	
SOA × Am × Res × Gen	2,30	1.50	0.09		0.42	0.03	
SOA × Am × Num × Gen	2,30	0.05	0.00		0.18	0.01	
SOA × Res × Num × Gen	2,30	2.10	0.12		1.57	0.09	
Am × Res × Num × Gen	1,15	0.35	0.12		0.04	0.00	
SOA × Am × Res × Num × Gen	2,30	0.25	0.02		2.57	0.15	

*SOA, stimulus-onset asynchrony; Am, action mode; Res, response; Num, number; Gen, gender. **p* < 0.05, ***p* < 0.01*.

### Experiment 2

#### Subtask 1

R1 was correctly initiated in the stimulus-based group in 269 ms, on average, and in the intention-based group in 331 ms, on average [*t*(30) = 3.72, *p* < 0.001, *d* = 1.32, two-tailed]. Errors of R1 in the stimulus-based group were rare (0.1%), as were anticipations and response omissions (stimulus-based: 0.6 and 0.7%, respectively; intention-based: 0.2 and 1.2%, respectively). The distribution of left-hand vs. right-hand key presses in intention-based trials was nearly equal (49.7 vs. 50.3%; average of absolute difference between left- and right-hand key presses = 2.9%) and provided a comparable amount of response repetitions and alternations.

#### Subtask 2

**Table A3:** **Means of mean reaction times (RTs, in ms) and error rates (ER) for R2 in Experiment 2 as a function of the relationship between S1 and S2 and between R1 and R2 and stimulus-onset asynchrony for intention-based and stimulus-based group**.

Stimulus feature repeated	Response							
	Intention-based				Stimulus-based			
	Repeated		Alternated		Repeated		Alternated	
	RT	ER	RT	ER	RT	ER	RT	ER
**SOA 1000 ms**								
Neither	600	6.9	538	1.5	691	10.9	560	0.8
Number	573	3.8	566	4.2	602	1.6	614	3.7
Gender	602	6.1	540	0.0	648	4.5	572	0.3
Both	510	2.8	597	8.8	542	0.5	618	9.5
**SOA 2000 ms**								
Neither	581	3.8	518	0.3	621	2.9	562	0.3
Number	573	4.9	547	2.7	591	1.6	579	1.1
Gender	574	8.3	546	0.3	622	3.7	572	0.5
Both	494	2.4	560	6.8	540	0.5	585	7.3
**SOA 6000 ms**								
Neither	558	3.3	507	1.8	583	2.9	540	0.3
Number	543	3.1	518	2.1	583	1.8	564	1.6
Gender	539	2.6	514	1.0	579	2.1	542	1.0
Both	511	3.5	528	2.2	541	0.0	561	2.8

*SOA, stimulus-onset asynchrony*.

**Table A4:** **Results of analysis of variance on mean reaction time of correct responses (RT) and error rates (ER) for Experiment 2**.

Effect	*df*	RT_R2_			ER_R2_		
		*F*	$\eta_{p}^{2}$	*p*	*F*	$\eta_{p}^{2}$	*p*
**BETWEEN SUBJECTS**							
Am	1, 30	1.40	0.04		1.60	0.05	
**WITHIN SUBJECTS**							
SOA	2, 60	14.57	0.33	**	17.95	0.37	**
Response (Res)	1,30	15.40	0.34	**	4.90	0.14	*
Number (Num)	1,30	9.29	0.24	**	1.98	0.06	
Gender (Gen)	1,30	19.25	0.39	**	3.76	0.11	
SOA × Am	2,60	0.08	0.00		1.34	0.04	
SOA × Res	2,60	0.23	0.01		0.11	0.00	
SOA × Num	2,60	3.47	0.10	*	0.79	0.03	
SOA × Gen	2,60	0.34	0.01		2.86	0.09	
Am × Res	1,30	0.62	0.02		2.21	0.07	
Am × Num	1,30	0.77	0.03		1.01	0.03	
Am × Gen	1,30	1.02	0.03		0.36	0.01	
Res × Num	1,30	90.43	0.75	**	53.15	0.64	**
Res × Gen	1,30	24.98	0.45	**	11.58	0.28	**
Num × Gen	1,30	15,81	0.35	**	7.79	0.20	**
SOA × Am × Res	2,60	0.93	0.03		1.30	0.04	
SOA × Am × Num	2,60	1.46	0.05		0.90	0.03	
SOA × Am × Gen	2,60	1.09	0.04		0.22	0.01	
SOA × Res × Num	2,60	26.94	0.47	**	25.44	0.46	**
SOA × Res × Gen	2,60	1.56	0.05		3.75	0.11	*
SOA × Num × Gen	2,60	1.65	0.05		7.30	0.20	**
Am × Res × Num	1,30	1.40	0.04		1.16	0.04	
Am × Res × Gen	1,30	0.30	0.01		3.72	0.11	
Am × Num × Gen	1,30	0.01	0.00		1.99	0.06	
Res × Num × Gen	1,30	16.61	0.36	**	17.19	0.36	**
SOA × Am × Res × Num	2,60	1.86	0.06		1.77	0.06	
SOA × Am × Res × Gen	2,60	1.13	0.04		0.23	0.01	
SOA × Am × Num × Gen	2,60	0.68	0.02		2.22	0.07	
SOA × Res × Num × Gen	2,60	0.61	0.02		5.85	0.16	**
Am × Res × Num × Gen	1,30	1.46	0.05		1.10	0.04	
SOA × Am × Res × Num × Gen	2,60	1.78	0.06		0.92	0.03	

*SOA, stimulus-onset asynchrony; Am, action mode; Res, response; Num, number; Gen, gender. **p* < 0.05, ***p* < 0.01*.

### Experiment 3

#### Acquisition phase – subtask 1

Participants of the stimulus-based group executed R1 correctly with a mean RT of 290 ms, whereas participants of the intention-based group executed freely selected actions with a mean RT of 334 ms [*t*(30) = 1.94, *p* = 0.06, two-tailed]. Errors of R1 in the stimulus-based group were rare (0.03%), as were anticipations and response omissions (stimulus-based: 0.4 and 0.8%, respectively; intention-based: 0.1 and 0.8% respectively). The distribution of left-hand vs. right-hand key presses in the intention-based group was nearly equal (49.4 vs. 50.6%, respectively; average of absolute difference between left- and right-hand key presses = 5.6%) which provided a comparable amount of response repetitions and alternations (49.6 vs. 50.4%).

#### Acquisition phase – subtask 2

**Table A5:** **Means of mean reaction times (RTs, in ms) and error rates (ER) for R2 in the acquisition phase of Experiment 3 as a function of the relationship between S1 and S2 and between R1 and R2 for intention-based and stimulus-based group**.

Stimulus feature repeated	Response							
	Intention-based				Stimulus-based			
	Repeated		Alternated		Repeated		Alternated	
	RT	ER	RT	ER	RT	ER	RT	ER
Neither	543	8.5	482	0.5	514	6.1	452	0.8
Number	534	3.7	557	4.2	494	3.7	510	4.0
Gender	544	5.9	495	2.1	524	3.9	473	0.8
Both	471	1.7	557	8.2	423	1.1	482	5.0
